# Left Ventricular Mechanics in Repaired Tetralogy of Fallot with and without Pulmonary Valve Replacement: Analysis by Three-Dimensional Speckle Tracking Echocardiography

**DOI:** 10.1371/journal.pone.0078826

**Published:** 2013-11-06

**Authors:** Shu-na Li, Wei Yu, Clare Tik-man Lai, Sophia J. Wong, Yiu-fai Cheung

**Affiliations:** Division of Paediatric Cardiology, Department of Paediatrics and Adolescent Medicine, Queen Mary Hospital, The University of Hong Kong, Hong Kong, China; University of Buenos Aires, Faculty of Medicine. Cardiovascular Pathophysiology Institute, Argentina

## Abstract

**Background:**

Altered septal curvature and left ventricular (LV) geometry secondary to right ventricular (RV) dilation render two-dimensional assessment of LV mechanics difficult in repaired tetralogy of Fallot (TOF) patients. The novel three-dimensional (3D) speckle tracking echocardiography enables comprehensive evaluation of true 3D LV mechanics.

**Methods and Results:**

Seventy-six patients aged 23.6±8.3 years, 55 with isolated repair (group I) and 21 with subsequent pulmonary valve replacement (group II), and 34 healthy controls were studied. Three-dimensional volume datasets were acquired for assessment of LV global and regional 3D strain, systolic dyssynchrony index (SDI), twist, twist gradient (twist/LV length), and ejection fraction. A global performance index was calculated as (global 3D strain•twist gradient)/SDI. The septal curvature and LV eccentricity were determined from the mid-ventricular short-axis. Compared with controls, group I and II patients had significantly reduced LV global 3D strain, LV twist, twist gradient, septal curvature, and global performance index, and greater LV systolic and diastolic eccentricity and SDI (all p<0.05). All but the four apical LV segments in patients had reduced regional 3D strain compared with controls (all p<0.05). Septal curvature correlated with LV global 3D strain (r = 0.41, p<0.001), average septal strain (r = 0.38, p<0.001), twist (r = 0.32, p<0.001), twist gradient (r = 0.33, p<0.001), and global performance index (r = 0.43, p<0.001).

**Conclusions:**

Adverse 3D LV mechanics as characterized by impaired global and regional 3D systolic strain, mechanical dyssynchrony, and reduced twist is related to reduced septal curvature in repaired TOF patients with and without pulmonary valve replacement.

## Introduction

Adverse left ventricular (LV) mechanics and its implications on long-term outcomes are increasingly recognized in patients after surgical repair of tetralogy of Fallot (TOF) [1], [2]. Impairment of longitudinal myocardial deformation has been demonstrated by one-dimensional tissue Doppler imaging [3], while disturbances of deformation in the circumferential and radial directions have been shown by two-dimensional speckle tracking echocardiography [4], [5]. Differences in timing of LV segmental volume changes further suggest systolic mechanical dyssynchrony after TOF repair [6], [7]. Assessment of LV mechanics by tissue Doppler imaging is nonetheless limited by angle dependence. While two-dimensional speckle tracking echocardiography allows assessment of deformation of three principal strain components [8], the through-plane motion of speckles remains an important inherent problem. Distortion of the left ventricle after TOF repair due to alteration of septal geometry and right ventricular (RV) volume overload render LV deformation analysis in two dimensions even more complex.

The newly introduced three-dimensional speckle tracking echocardiography (3DSTE) allows simultaneous imaging of all LV segments and tracking of speckle motion vectors in three dimensions based on the full volume dataset [9]–[11]. Furthermore, as segments of LV apex and base are imaged simultaneously, LV torsion can be assessed from a single acquisition. This technique has recently been validated against sonomicrometry in the quantification of LV myocardial strain and torsion in animal models [12] and shown to be useful in the quantification of dyssynchrony in adults with heart failure [13].

The aim of this study was to assess comprehensively the LV mechanics in terms of true 3D systolic strain, twist, and dyssynchrony in patients after TOF repair with and without pulmonary valve replacement (PVR) with the new 3DSTE. We explored further relationships between 3D LV myocardial mechanics and ventricular septal curvature and LV geometry and the application of a new 3D global performance index.

## Methods

### Ethics statement

All patients and parents of minors gave written informed consent to participate in this study approved by the Institutional Review Board of The University of Hong Kong/Hospital Authority West Cluster, Hong Kong.

### Subjects

Patients who had undergone total surgical repair of TOF with and without additional PVR were recruited from the congenital heart clinic. In our centre, PVR for significant pulmonary regurgitation was performed in symptomatic patients with heart failure and/or exercise intolerance and in asymptomatic patients with progressive or moderate to severe RV dilation (>180 ml/m^2^ as assessed by cardiac magnetic resonance). The following patient data were collected: demographics, age at operations, and duration of follow-up since surgical repair of TOF and PVR. Healthy subjects were recruited as controls. These healthy controls were healthy volunteers, healthy siblings of patients, and subjects followed up for non-specific chest pain for which no organic causes were identifiable. The body weight and height of all subjects were measured and the body surface area was calculated accordingly.

### Two-dimensional and colour Doppler echocardiography

Echocardiographic imaging was performed with the Artida ultrasound system (Toshiba Medical Systems, Tokyo, Japan). All echocardiographic recordings were stored in an external hard disc for offline analyses. The average of 3 measurements was used for statistical analyses.

From the apical 4-chamber view, the RV end-diastolic and RV end-systolic areas were traced. The RV fractional area change was calculated as (end-diastolic area minus end-systolic area)/end-diastolic area. Severity of pulmonary and tricuspid regurgitation was graded semi-quantitatively by colour Doppler imaging [14].

**Figure 1:**
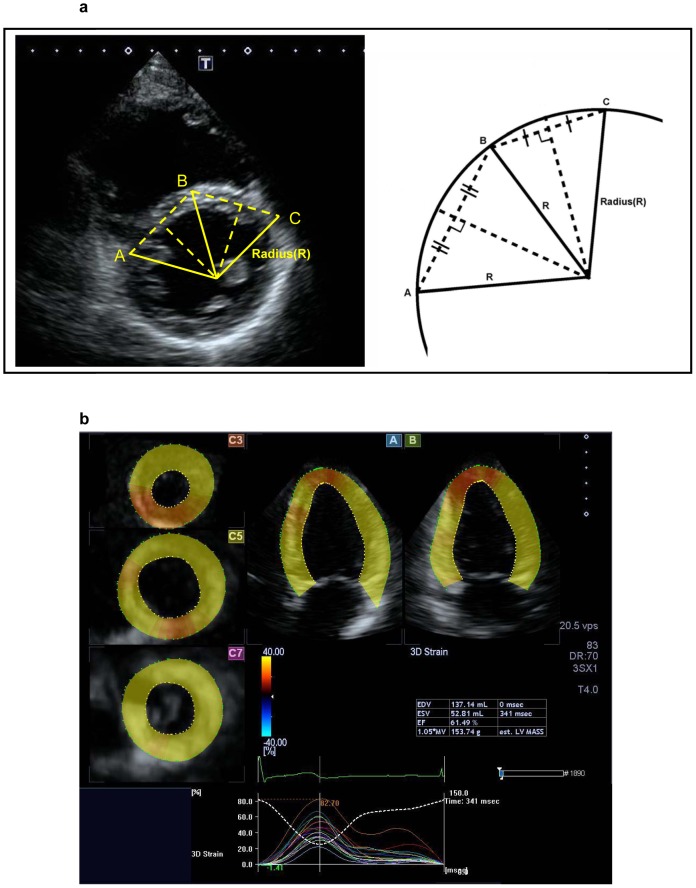
Two-dimensional echocardiographic assessment of ventricular septal configuration and 3D speckle tracking analysis. (a) The radius was identification from the intersection of perpendicular bisectors between the anterior (C) and middle (B) and between the posterior (A) and middle (B) points in the ventricular septum. (b) The 5-plane view used for 3D speckle tracking analysis: A plane (apical 4-chamber view), B plane (2-chamber view), and 3 C planes (short-axis views near the apex, at mid-level, and at the base of the left ventricle).

The ventricular septal configuration was quantified from the parasternal mid-short axis view at end-diastole and expressed as curvature, the reciprocal of radius [15]. Briefly, three points were marked at the anterior, middle, and posterior aspects of the septum. Intersection of the perpendicular bisectors between the anterior and middle and between the posterior and middle points was determined and the radius was measured (Fig. 1a). The septal curvature was calculated as 1/radius (in cm). A positive value was assigned for physiologic rightward curvature and a negative value for leftward curvature.

Eccentricity of the left ventricle was assessed from the parasternal mid short-axis view at papillary muscle level. Distance from the endocardial surface of the mid-ventricular septum to that of the posterior-lateral LV free wall (D1) and distance of the orthogonal axis between endocardial surfaces of the anterior and inferior LV free walls (D2) were measured at end-systole and end-diastole. The systolic and diastolic LV eccentricity index (EI) was calculated as D2/D1 measured respectively at end-systole and end-diastole [16].

### Three-dimensional speckle tracking echocardiographic assessment

Real-time 3D echocardiographic imaging was performed from the apical view using a matrix array transducer interfaced to the Artida ultrasound system (Toshiba Medical Systems, Tokyo, Japan). Full-volume acquisition with capturing of 4 sub-volumes over 4 consecutive cardiac cycles was performed during breathhold. A wide sector width (80–90 degrees by 80–90 degrees) was chosen and care was taken to include the entire LV cavity during acquisition. The settings were optimized to ensure a volume rate of 20 to 30 volumes per second.

The volume datasets were analyzed using the 3D wall motion tracking software (Toshiba Medical Systems, Tokyo, Japan). From the three-dimensional dataset, three planes were generated: i) apical four-chamber view (A plane), ii) an orthogonal two-chamber view (B plane), and iii) three short axis views near the apex, at mid-level, and at the base of the left ventricle (C planes) (Fig. 1b). In the A and B planes, three points of the endocardium were marked manually, one at the apex and the two at the edges of the mitral valve ring. The LV endocardium and epicardium were then traced automatically by the software, and verified and adjusted manually where necessary based on all views of the three planes.

Based on tracking of the endocardial surface throughout the cardiac cycle, the LV systolic and diastolic volumes and LV ejection fraction were calculated. Based on the tracking of motion vectors of speckles in three dimensions between the endocardium and epicardium, the global systolic 3D strain of the entire left ventricle and other 3DSTE parameters were derived. The software automatically divided the left ventricle into 16 segments as defined by the American Society of Echocardiography [17]. The regional 3D strain of each of the segments was determined accordingly. Left ventricular dyssynchrony was quantified by calculation of the 3DSTE-derived systolic dyssynchrony index (SDI), which represents the standard deviation of the times to peak 3D strain of the 16 segments expressed as a percentage of RR interval [18]. The LV twist was derived from the systolic rotation of the apical segments relative to the base [19]. The LV twist was further divided by the distance of apical segments from the base (LV twist gradient) to adjust for potential differences in LV dimensions between patients and controls.

The intra- and interobserver variability of 3DSTE parameters was assessed in 10 patients and 10 control subjects and reported as the coefficients of variation, calculated by dividing the SD of the differences between measurements by the mean and expressed as a percentage. The intraobserver variability for LV 3D global strain, SDI, twist, and twist gradient was 8.0%, 8.1%, 7.8%, and 8.9%, respectively; while the interobserver variability was 10.8%, 10.4%, 9.2%, and 10.7%, respectively.

### Assessment of 3D LV global performance

A 3D LV global performance plot was generated by plotting onto the x-, y-, and z-axis of respectively peak global 3D strain, LV twist gradient, and reciprocal of LV SDI. A 3D LV global performance index was calculated as (global 3D strain•twist gradient)/SDI.

### Statistical analysis

Data are expressed as mean±standard deviation. The RV area and LV volume data were normalized by body surface area. The absolute values of 3D strain were used to facilitate interpretation and analyses. Differences in demographic and echocardiographic parameters among patients without PVR, patients with PVR, and controls were compared using simple analysis of variance (ANOVA), while comparisons between two groups were performed using unpaired Student's t test. Pearson correlation analysis was used to assess for relationships between septal curvature, LV eccentricity, and parameters of LV mechanics with Bonferonni adjustment for multiple correlations. The area under the receiver operating characteristic (ROC) curve was calculated to determine capability of GPI to discriminate between patients and controls. A two-tailed p value <0.05 was considered statistically significant. All statistical analyses were performed using SPSS 15.0 (SPSS, Chicago, Illinois, USA).

## Results

### Subjects

Of the total of 114 subjects recruited, 110 (96%) subjects had optimal echocardiographic images for analysis, including 76 patients and 34 controls. The 76 patients were aged 23.6±8.3 years and had undergone TOF repair at 4.9±3.7 years. They were studied at 18.8±7.4 years after initial repair. Of the 76 patients, 55 had only isolated TOF repair (group I), while 21 had additional PVR (group II). The PVR was performed at the age of 23.1±6.9 years, and the patients were studied at 2.2±2.5 years after PVR. In group I, 5 (9%) patients were on cardiac medications, which were beta-blockers in 2, angiotensin-converting enzyme inhibitor or angiotensin receptor blocker in 2, diuretics in 1, and digoxin in 1. In group II, 7 (33%) patients were on medications, which included anti-platelet agent or anticoagulants in 6, diuretics in 2, angiotensin-converting enzyme inhibitor in 2, beta-blocker in 1, and digoxin in 1. Thirty-four controls (group III) aged 20.9±7.5 years were studied. Comparisons of demographic and echocardiographic variables among the three groups are summarized in Table 1.

**Table 1:** Demographic and echocardiographic parameters.

	Group I	Group II	Group III	ANOVA	p1	p2	p3
	(n = 55)	(n = 21)	(n = 34)	p	(I vs III)	(II vs III)	(I vs II)
*Demographic data*							
Age	23.1±8.9	24.8±6.7	20.9±7.5	0.21			
Sex (M/F)	33/22	15/6	18/16	0.40**			
Body weight(kg)	55.0±18.1	53.5±11.5	49.9±14.0	0.34			
Body surface area(m^2^)	1.5±0.3	1.6±0.2	1.5±0.3	0.59			
*RV parameters*							
End-systolic area (cm^2^/m^2^)	12.2±5.1	10.6±4.1	4.7±1.4	<0.001*	<0.001*	<0.001*	0.21
End-diastolic area (cm^2^/m^2^)	19.8±6.5	16.7±4.1	9.3±2.0	<0.001*	<0.001*	<0.001*	0.05*
Fractional area change (%)	41.5±9.0	38.7±10.0	50.6±10.6	<0.001*	<0.001*	<0.001*	0.24
*LV and septal geometric indices*							
End-systolic volume (ml/m^2^)	30.8±8.9	33.4±9.6	24.5±6.2	<0.001*	<0.001*	<0.001*	0.27
End-diastolic volume (ml/m^2^)	57.9±14.9	65.6±16.2	58.1±14.2	0.11	0.94	0.76	0.05
Ejection fraction (%)	46.7±6.1	49.2±6.2	57.7±4.8	<0.001*	<0.001*	<0.001*	0.12
End-systolic EI	1.19±0.11	1.18±0.12	1.1±0.04	<0.001*	<0.001*	<0.001*	0.59
End-diastolic EI	1.29±0.16	1.22±0.11	1.05±0.04	<0.001*	<0.001*	<0.001*	0.02*
Septal curvature	0.23±0.08	0.25±0.08	0.33±0.04	<0.001*	<0.001*	<0.001*	0.27
*Indices of LV mechanics*							
LV 3D global strain (%)	27.5±7.1	26.3±6.9	39.8±7.2	<0.001*	<0.001*	<0.001*	0.53
Average septal 3D strain (%)	30.6±9.5	26.0±8.3	47.7±9.1	<0.001*	<0.001*	<0.001*	0.05
SDI (%)	10.3±3.8	9.5±3.8	5.6±2.6	<0.001*	<0.001*	<0.001*	0.41
LV twist (degree)	6.27±2.60	6.88±2.57	10.69±3.73	<0.001*	<0.001*	<0.001*	0.36
LV twist gradient (degree/cm)	1.16±0.56	1.19±0.46	2.00±0.85	<0.001*	<0.001*	<0.001*	0.87

Abbreviations: 3D, three-dimensional, EI, eccentricity index, LV, left ventricular, SDI, systolic dyssynchrony index.

* Statistically significant.

** Compared using Chi-squared test.

### Conventional echocardiographic parameters

Compared with controls, both group I and II patients had significantly larger RV end-systolic and end-diastolic areas and LV end-systolic volume and reduced RV fractional area change and LV ejection fraction (all p<0.05) (Table 1).

In group I, pulmonary regurgitation was moderate-to-severe in degree in 22 patients, mild in 25, and absent in 8, while in group II, the regurgitation was moderate in 3, mild in 6, and absent in 12. All patients had absent or trivial degree of tricuspid regurgitation except for 5 group I patients who had mild to moderate degree of regurgitation. Compared with group I patients, group II patients tended to have smaller RV end-diastolic area (p = 0.05), but similar RV factional area change (p = 0.24) and LV ejection fraction (p = 0.12).

### 3D myocardial deformation and twist

**Figure 2:**
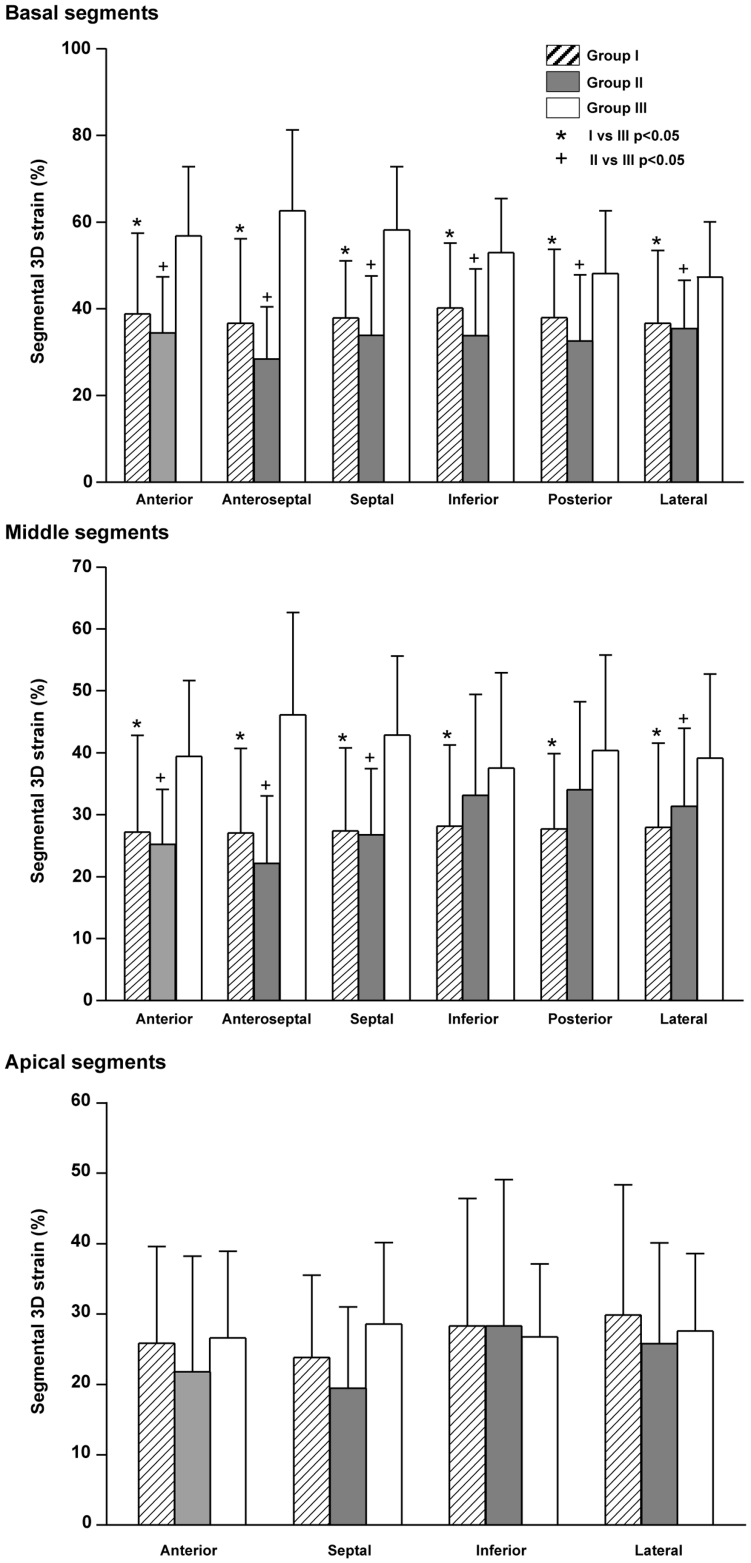
Regional 3D strain of the basal, middle, and apical segments in group I and II patients and group III control subjects.

The global LV 3D strain was significantly lower in group I and II (both p<0.001) patients than controls (Table 1). Figure 2 shows the regional 3D strain in the three groups. Group I and II patients had reduced regional 3D strain of all but the four apical segments.

The average strain of the 5 septal segments in group I patients was 66.0±10.1% of the average values in controls, and tended to be lower than the 81.4±15.0% (p = 0.056) for the 11 non-septal segments. The average strain of the 5 septal segments in group II patients was 56.6±9.3% of the average values in controls, being significantly lower than the 78.6±14.1% (p = 0.007) for the 11 non-septal segments.

**Figure 3:**
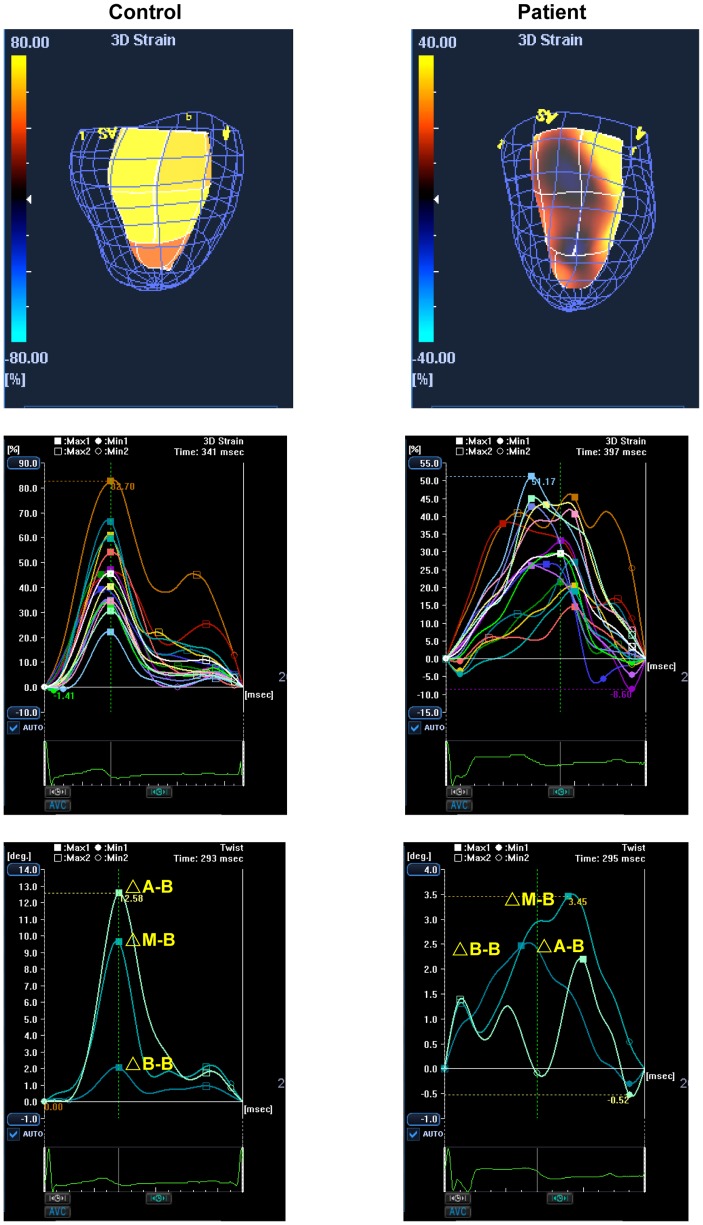
Representative 3D strain images, 3D strain curves and twist curves in a control and a patient with repaired TOF. Heterogeneous colour-coded segmental 3D strain (upper panel), reduced 3D strain and wider dispersion of time to peak 3D strain (middle panel) and reduced LV twist (△A-B) (lower panel) are illustrated in the curves derived from the patient. (△A-B, difference in rotation between left ventricular apical and base at attachments of the mitral valve, △M-B, between mid-segments and base, and △B-B between basal segments and base)

As for synchronicity of the timing to peak 3D strain, the 3DSTE-derived SDI was significantly greater in both group I (p<0.001) and II (p<0.001) patients than controls. On the other hand, both the LV twist and twist gradient were significantly lower in group I and II patients than controls (all p<0.001). Figure 3 shows examples of regional 3D strain-time curves and LV twisting curves in a patient and a control subject.

Compared with group I patients, group II patients did not show significant differences in 3D strain, SDI, LV twist, and LV twist gradient despite PVR (all p>0.05) (Table 1).

For the whole cohort, global 3D strain correlated negatively with SDI (r = −0.59, p<0.001), RV end-systolic area (r = −0.48, p<0.001), and RV end-diastolic area (r = −0.47, p<0.001), but positively with LV twist (r = 0.31, p = 0.001), LV twist gradient (r = 0.28, p = 0.003), LV ejection fraction (r = 0.62, p<0.001), and RV fractional area change (r = 0.42, p<0.001).

### 3D LV global performance

**Figure 4:**
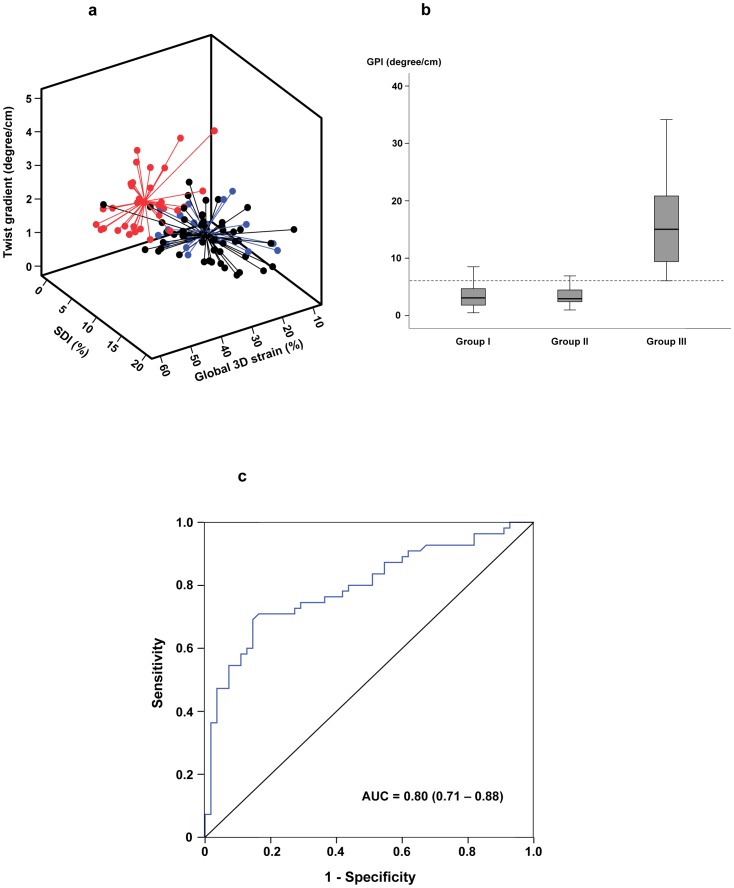
Assessment of left ventricular function by the global performance index (GPI). (a) The 3D global performance plot showing different left ventricular performance in group I patients (black), group II patients (blue), and controls (red). The centre point for each group represents the mean of the values in the x-, y-, and z-axes. (b) Box-plots showing the GPI in the three groups of subjects. The dotted line represents the 10^th^ centile line derived from normal control data. (c) The receiver–operator characteristic curve for GPI to discriminate between patients (group I and II) and controls. AUC, area under curve.

The 3D global performance plot, which took into account global 3D strain, SDI, and LV twist adjusted for LV length is shown in Figure 4a. The 3D global performance index was significantly lower in group I (p<0.001) and II (p<0.001) patients compared with controls (Fig. 4b). There was no difference in global performance index between group I and II patients (p = 0.78).

The lower limit of control 3D global performance index was 6.07 degree/cm, derived from mean minus 1.28 SD and corresponded to the 10^th^ percentile limit. Using this as the lower limit of normal, 85.5% (47/55) of group I patients and 90.0% (17/21) of group II patients had impaired 3D global LV performance. For the ROC analysis, the best GPI cutoff to discriminate between patients and controls was 5.79 (deg/cm), which had a sensitivity of 0.71 and specificity of 0.84 (Fig. 4c). The area under curve of the ROC curve is 0.80 with a 95%CI of 0.71 to 0.88 (p<0.001).

### Septal curvature, LV geometry and myocardial mechanics

The septal curvature of group I and II patient was both significantly smaller than controls (p<0.001), while both the systolic and diastolic EIs were significantly greater in group I and II patients than controls (all p<0.001) (Table 1). The septal curvature correlated negatively with RV end-systolic area (r = −0.48, p<0.001) and end-diastolic area (r = −0.53, p<0.001).

Table 2 shows the correlations between septal curvature, EIs and indices of LV mechanics. For the whole cohort, septal curvature correlated positively with global 3D strain, average 3D strain of the 5 septal segments, LV twist, LV twist gradient and global performance index (all p≤0.001). Additionally, the LV diastolic EI was associated with all the 3DSTE parameters (all p≤0.002).

**Table 2:** Correlations between septal curvature and left ventricular eccentricity and indices of three-dimensional left ventricular mechanics.

	Septal curvature		Systolic EI		Diastolic EI	
	r	p	r	p	r	p
3D global strain	0.41	<0.001*	-0.44	<0.001*	-0.46	<0.001*
Average septal 3D strain	0.38	<0.001*	-0.38	<0.001*	-0.41	<0.001*
SDI	-0.29	0.003	0.36	<0.001*	0.45	<0.001*
LV twist	0.32	0.001*	-0.20	0.039	-0.32	0.001*
LV twist gradient	0.33	<0.001*	0.17	0.082	-0.30	0.002*
Global performance index	0.43	<0.001*	0.41	<0.001*	-0.50	<0.001*

Abbreviations: 3D, three-dimensional, EI, eccentricity index, LV, left ventricular, SDI, systolic dyssynchrony index.

* Statistically significant after Bonferroni adjustments.

## Discussion

To our knowledge, this is the first study to explore the usefulness of 3DSTE in the interrogation of LV mechanics in repaired TOF patients with and without PVR. Novel findings of the present study include 1) adverse 3D LV mechanics as characterized by impaired global and regional 3D systolic strain, mechanical dyssynchrony, and reduced twist adjusted for LV length in repaired TOF patients with significant pulmonary regurgitation and even in those after PVR, 2) strong associations between septal curvature and 3D indices of LV mechanics, and 3) potential usefulness of 3D global performance index in the comprehensive evaluation of LV dysfunction in patients.

Characterization of LV mechanics in patients after TOF repair has been limited to the use of one-dimensional angle-dependent tissue Doppler imaging [3] and two-dimensional strain imaging [4]. The latter, while overcoming some of the limitations of Doppler techniques, does not allow simultaneous analyses of all LV segments and tracking of out-of-plane speckle motions. Furthermore, alteration of septal geometry, paradoxical systolic septal motion, and the eccentric LV geometry secondary to RV volume overload in repaired TOF complicate two-dimensional assessment of LV mechanics. By contrast, the recently validated 3DSTE has the advantages of being enable to acquire data and to analyze regional and global motion of all LV segments with a single acquisition of four consecutive cardiac cycles, and to track speckle motions in genuinely three dimensions [9]–[13]. In our cohort of subjects, quality data could be obtained for 3D wall motion tracking analysis in 96% (110/114) with reasonably low intra- and interobserver variability in the analysis of 3DSTE parameters.

The significantly lower global 3D strain in our patients is contributed primarily by reduction of basal and mid LV segmental 3D strain (Figure 2), while the apical segmental 3D strain is relatively preserved. Whereas reduced LV apical longitudinal strain has been documented in repaired TOF patients [3], [20], [21], increased apical systolic wall thickening and shortening fraction have been demonstrated respectively by cardiac magnetic resonance [22] and cine-angiography [23]. Although the explanation of the this compensatory apical hypercontractility is unclear, our finding is consistent with compensatory increase in either the radial or circumferential strain or both to account for the normal apical 3D strain in patients regardless of severity of pulmonary regurgitation. On the other hand, the non-contractile patch used for the repair of ventricular septal defect and loss of myocardium may explain the reduced deformation of the basal septum. Additionally, the patch may potentially also contribute to impaired torsional mechanics found in repaired TOF patients.

Dispersion of the times to peak 3D strain of different LV segments further provides convenient quantification of LV systolic mechanical dyssynchrony. Exploration and quantification of LV dyssynchrony in repaired TOF patients have previously been based on tissue Doppler imaging [20], two-dimensional speckle tracking [1], and segmental volume-derived SDI [6], [7]. The volume-derived SDI, while being a 3D quantitative approach, does not however directly assess deformation of myocardium, hence failing to differentiate active myocardial contraction from passive tethering of adjacent fibrous tissue. The present study fills these gaps by directly interrogating active systolic myocardial deformation of all LV segments in a truly 3D manner. Indeed, the usefulness of 3DSTE to quantify LV dyssynchrony and to identify the site of latest mechanical activation has recently been shown in adults with heart failure undergoing cardiac resynchronization therapy [13].

Simultaneous acquisition of LV apical and basal motion minimizes the errors in the calculation of LV twist and twist gradient secondary to differences in heart rate when the apical and basal planes are acquired separately. The understanding of torsional mechanics in repaired TOF patients is limited. Our findings agree with the few published reports that showed a reduction of LV systolic apical rotation and twist in these patients [21], [24], [25]. We showed additionally that normalization of the magnitude of twist to LV length, which has been shown to allow comparability of LV twist of hearts of different sizes across species [26], revealed similarly differences between patients with or without PVR and control subjects. Importantly, we have further found that LV twist and twist gradient are related inversely to septal curvature.

There is a paucity of data on the relationship between alteration in septal geometry and LV dysfunction in repaired TOF patients. The ventricular septum has been regarded as a key element for ventricular systolic and diastolic ventricular interaction.[27] Septal excursion, an alternative method to assess septal geometry using cardiac magnetic resonance, has been shown to be increased in repaired TOF patients with RV volume overload.[28] Importantly, increased septal excursion has been associated with more prominent LV fibrosis and reduced LV ejection fraction [28]. In the present study, we further found that 3D strain was reduced to a greater extent in septal than non-septal segments in both patients with and without PVR. Additionally, moderately strong associations were found between septal curvature and indices of 3D LV mechanics. Taken together, it is tempting to speculate that alteration of septal geometry may be an important contributor of impaired LV mechanics and function in repaired TOF. It is worthwhile emphasizing, however, the multifactorial causes of LV dysfunction in repaired TOF patients. Hence, apart from adverse ventricular-ventricular interaction mediated possibly septal distortion, pre-operative hypoxaemia, perioperative myocardial injury, and post-operative mechanical dyssynchrony have also been hypothesized [6], [7], [29]–[31]. The latter is also evidenced by the greater dispersion of times to peak 3D strain of different LV segments in our patients. With regard to the relationship between septal curvature and LV twist and twist gradient, the contribution of septum to LV twisting is supported by animal studies that reveal the oblique architecture of the septal myocardium [32].

To evaluate in a comprehensive manner the LV mechanics in repaired TOF patients, we used the simultaneously acquired LV indices to derive the 3D global performance plot and index. Our proposed 3D global performance plot incorporates the three important parameters of LV mechanics (Fig. 4a). This index, by taking into account of the third dimension, may supercede our previously reported global index based on two-dimensional area strain and SDI [33]. Based on normal data, the lower reference limit of global performance index can be defined and may be utilized to unmask LV dysfunction in patients who would otherwise be regarded as normal based on measurement of LV ejection fraction. Serial tracking of LV status using the 3D plot may be useful for prognostication and to assess the timing and impact of PVR, although this remains speculative. Indeed, LV longitudinal deformation indices have recently been shown to predict adverse outcomes including sudden cardiac death and life-threatening ventricular arrhythmias in repaired TOF patients [34]. The prognostic significance of prolonged QRS duration [35] and RV dilation and dysfunction [2], [30] and the importance of long-term regular electrocardiographic assessment and monitoring of RV function by cardiac magnetic resonance for determining the optimal timing of PVR are well known.[36] Increasingly, the importance of assessing LV function in determining the timing of PVR has also been recognized [36]–[38].

It is uncertain whether PVR has an impact on the altered LV mechanics and long-term outcomes. Nonetheless, our preliminary data based on a relatively small cross-sectional cohort of young adults with surgical PVR revealed similarly impaired LV 3D mechanics as those with uncorrected pulmonary regurgitation. It is intriguing that the septal curvature and RV fractional area change remained similar after PVR. The latter agrees with our previous meta-analysis that revealed the absence of significant changes in RV ejection fraction despite surgical PVR [39]. Our findings are also in agreement with those of Frigiola et al who failed to demonstrate improvement in relatively load-independent indices of LV and RV function in adolescent and young adult TOF patients after surgical PVR [40]. A recent study also showed the absence of further positive LV or RV remodeling beyond the acute effects of percutaneous intervention in patients undergoing percutaneous PVR [41].

Several limitations to this study require comments. Firstly, the number of patients who had undergone PVR was relatively small in our institution. It would have been ideal to monitor the indices of 3D mechanics in a larger cohort longitudinally to track the changes before and after implantation of the pulmonary valve. Secondly, the evaluation of septal curvature remains a cross-sectional and two-dimensional one. Serial longitudinal data would provide additional information on whether alteration of septal curvature is a static, reversible, or progressive phenomenon. Nonetheless, a recent study showed decrease in maximal septal excursion, as assessed by cardiac magnetic resonance, concomitant with reduction of RV volumes after PVR in repaired TOF patients, suggesting the possibility of septal remodeling with removal of RV volume overload [42]. Quantification of 3D septal curvature [43] and bilayer analysis of the ventricular septum [44] may shed even more lights on the impact of altered geometry of the basal and apical septum on LV deformation. Thirdly, the 3D volume frame rate is relatively low at 20 to 30 volumes per second. Nonetheless, quality data have been obtained in adults with heart failure for quantification of LV dyssynchrony [13]. The sacrifice of temporal resolution for spatial resolution may enhance speckle tracking.

In conclusion, this study implicates an influence of septal curvature on LV 3D mechanics and provides the first evidence of potential usefulness of 3DSTE in the comprehensive evaluation of LV mechanics in repaired TOF patients both before and after PVR. Further longitudinal studies are warranted to evaluate the impact of PVR on septal remodeling and LV mechanics.
